# Accumulation of Phenolic Compounds and Expression Profiles of Phenolic Acid Biosynthesis-Related Genes in Developing Grains of White, Purple, and Red Wheat

**DOI:** 10.3389/fpls.2016.00528

**Published:** 2016-04-22

**Authors:** Dongyun Ma, Yaoguang Li, Jian Zhang, Chenyang Wang, Haixia Qin, Huina Ding, Yingxin Xie, Tiancai Guo

**Affiliations:** ^1^Agronomy/National Engineering Research Center for Wheat, Henan Agricultural UniversityZhengzhou, China; ^2^The Collaborative Innovation Center of Henan Food Crops, Henan Agricultural UniversityZhengzhou, China; ^3^Food and Science Technology College, Henan Agricultural UniversityZhengzhou, China; ^4^The National Key Laboratory of Wheat and Maize Crop Science, Henan Agricultural UniversityZhengzhou, China

**Keywords:** phenolic acid accumulation, gene expression, different-colored grains, wheat

## Abstract

Polyphenols in whole grain wheat have potential health benefits, but little is known about the expression patterns of phenolic acid biosynthesis genes and the accumulation of phenolic acid compounds in different-colored wheat grains. We found that purple wheat varieties had the highest total phenolic content (TPC) and antioxidant activity. Among phenolic acid compounds, bound ferulic acid, vanillic, and caffeic acid levels were significantly higher in purple wheat than in white and red wheat, while total soluble phenolic acid, soluble ferulic acid, and vanillic acid levels were significantly higher in purple and red wheat than in white wheat. Ferulic acid and syringic acid levels peaked at 14 days after anthesis (DAA), whereas *p*-coumaric acid and caffeic acid levels peaked at 7 DAA, and vanillic acid levels gradually increased during grain filling and peaked near ripeness (35 DAA). Nine phenolic acid biosynthesis pathway genes (*TaPAL1, TaPAL2, TaC3H1, TaC3H2, TaC4H, Ta4CL1, Ta4CL2, TaCOMT1*, and *TaCOMT2)* exhibited three distinct expression patterns during grain filling, which may be related to the different phenolic acids levels. White wheat had higher phenolic acid contents and relatively high gene expression at the early stage, while purple wheat had the highest phenolic acid contents and gene expression levels at later stages. These results suggest that the expression of phenolic acid biosynthesis genes may be closely related to phenolic acids accumulation.

## Introduction

Phenolic acids can be found in many plant species. These compounds play diverse roles, functioning as signaling molecules, agents in plant defense, and regulators of auxin transport. Additionally, these compounds have received increasing attention due to their antioxidant activity and free radical scavenging ability, which function in degenerative disease prevention. Most studies of plant phenolics have focused on examining these compounds in vegetables and fruits (Nile and Park, 2014; Neacsu et al., 2015). Many phenolic compounds in fruits and vegetables are also found in cereals (Adom and Liu, 2002; Acosta-Estrada et al., 2014). The total antioxidant activity of whole-grain products is similar to that of fruits or vegetables on a per serving basis (Wu et al., 2004), and regular consumption of whole grain foods helps to reduce the risk of cardiovascular disease, ischemic stroke, and type 2 diabetes (Lutsey et al., 2007).

Wheat (*Triticum aestivum*) is a staple food crop worldwide. Several studies have investigated the antioxidant activities of different types of wheat grains, as well as their phenolic profiles. Phenolic acids in cereals exist in free, soluble conjugate, and insoluble bound forms, primarily the latter. Adom and Liu (2002) reported that ferulic acid is the major phenolic compound in grains, with free, soluble-conjugated, and bound ferulic acids present at a 0.1:1:100 ratio. Bound phenolics are considered to have unique antioxidant and anti-inflammatory properties. These compounds help to reduce the risk of colorectal cancer because they can survive digestion in the stomach and small intestine, and they provide site-specific health benefits in the colon and other tissues after absorption (Liu, 2007). Okarter et al. (2010) found that bound phenolic contents in six diverse varieties of whole wheat range from 5.82 to 6.62 μmol gallic acid equivalents per gram, and the bound fraction contributes 53.8–69.7% of the total phenolic content (TPC). Phenolic acids are rich in the bran fraction, and their components are significantly influenced by the milling extraction rate (Wang et al., 2013). Siebenhandl et al. (2007) reported that TPC is higher in the bran fraction of purple wheat (822.23 mg/100 g) and blue wheat (761.64 mg/100 g) than in the corresponding flour fractions from purple and blue wheat (81.16 mg/100 g and 64.64 mg/100 g, respectively). Using gradual milling, Hung et al. (2009) found that the contents of free and bound phenolic extracts gradually increased from the inner to the outer fractions of wheat grain. Commercial white wheat flour, which lacks most of the bran and germ, lacks phenolic compounds. Thus, gradual milling and roller milling have been used to increase the amounts of phenolic compounds (Beta et al., 2005).

Recently, pigmented wheat has attracted increasing attention due to its high nutrient density. Purple- and blue-grained wheat have much higher free radical scavenging activity than white wheat. This antioxidant activity is positively correlated with the TPC of bran and whole wheat from these varieties (Li et al., 2005). Similar findings were also reported in rice, whose pericarp color is related to the concentration of phenolics in the grain, which is usually higher in varieties with red or black pericarps (Goffman and Bergman, 2004). Liu et al. (2010) reported that purple wheat varieties (Charcoal and Konini) have the highest and second highest phenolic contents, followed by red (Red Fife) and yellow (Luteus) wheat. We previously found that the average TPC of colored wheat is higher than that of white wheat (Ma et al., 2014).

The grain filling stage is an important step in the process of grain development and polyphenol accumulation. Knievel et al. (2009) found that anthocyanin levels in wheat increase rapidly during grain development and decrease before maturity. Žofajová et al. (2012) reported that different genotypes respond differently to the dynamics of total anthocyanin accumulation during grain filling. The highest TPC in white and red rice was detected 1 week after flowering, while black rice has the highest TPC at maturity (Shao et al., 2014). All phenolic acids in wheat grains are primarily derived from the phenylpropanoid biosynthetic pathway, which begins with the conversion of phenylalanine to cinnamic acid by phenylalanine ammonia lyase (PAL). McCallum and Walker (1990) found that PAL activity was maximal during the early milk stage of development and then declined, and PAL activity in red wheat was higher during early grain development. Qu et al. (2013) and Jiang et al. (2013) reported a relationship between phenolic compound accumulation and related enzyme activity/gene expression in rapeseed and tea, respectively.

Interest in the health benefits of whole grains has driven breeding programs and the development of cultivation practices aimed at further enhancing nutrient density in the grain. However, to date, the accumulation of phenolic acids and the expression patterns of phenolic acid biosynthesis genes in developing wheat grain have not been investigated. In this study, we investigated the expression profiles of phenolic acid biosynthesis genes in developing grains of white, red, and purple wheat via real-time PCR. The accumulation of phenolic acids was also examined. The results of this study provide a better understanding of phenolic acid biosynthesis in wheat grains.

## Materials and methods

### Experimental design

The seeds of six wheat (*Triticum aestivum* L.) varieties with different grain color (**Table 2**) were sown in 2013–2014 at the Henan Agricultural University Experimental Station, Zhengzhou, Henan province (34°44′N, 113°42′E). The experiment was laid out as a randomized block design with three replicates. The plot dimension was 3 m × 7 m. Seeds were sown on 16 October 2013, and the sowing density was 177 seeds/m^2^. Each plot received the same amount of N/P/K (112.5, 60, 150 kg/ha) before sowing. At the elongation stage, N (112.5 kg/ha) fertilization in the form of urea was used for top-dressing at each plot. Irrigating was applied to each plot at the elongation stage and flowering stage, respectively. Each time, the irrigation rate was 75 mm. The environmental conditions at the test sites are listed in the Supplementary Material (Supplementary Table S1). At flowering, spikes undergoing anthesis on the same day were tagged. The grains were harvested within 7-day intervals, starting from 7 days after anthesis (DAA) until seed maturation. The plant tissues were immediately frozen in liquid nitrogen and stored at −80°C until use. Mature grains were harvested by hand and cleaned before milling. Broken, shriveled, and foreign grains were removed by hand. The wheat grains were ground in a Cyclotec Sample Mill (Foss Tecator AB, Höganäs, Sweden).

### Determination of total phenolic content

Total phenolic compounds were extracted from the samples using the method reported by Shen et al. (2009), with slight modifications. Briefly, flour samples (2.0 g) were mixed with 16 mL of methanol containing 1% HCl for 24 h at 24°C. The procedure was repeated twice. The methanol extracts were centrifuged at 4000 × g for 15 min; the resulting supernatants were pooled and stored at 4°C. Extracts (0.5 mL) were mixed with 5 mL Folin Ciocalteu reagent, neutralized with 4 mL saturated sodium carbonate (75 g/L), and incubated at room temperature for 2 h. Absorbance at 765 nm was measured in a spectrophotometer. TPC is expressed as milligrams of ferulic acid equivalent (FAE) per gram of sample.

### Determination of phenolic acid composition

#### Sample preparation

Soluble-conjugated phenolic acid compounds were extracted according to the procedure described by Htwe et al. (2010) with minor modifications. Briefly, ground wheat samples were extracted with 20 mL of methanol: acetone: water (7:7:6, v/v/v) at room temperature and centrifuged (5000 × g, 15 min). The concentrated supernatants were treated with 1.5 mL of 2 M NaOH for 4 h at 50°C, and the pH was adjusted to 2.0 (6 M HCl). The mixture was centrifuged at 5000 × g for 15 min. The supernatant was extracted three times with diethyl ether/ethyl acetate (1:1, v/v), and the combined extract was evaporated to dryness at 30°C. To measure insoluble bound phenolic acid compounds, the precipitates were treated with 10 mL of 2 M NaOH for 12 h at room temperature. The samples were flushed with nitrogen and acidified to pH 2 with hydrochloric acid, followed by centrifugation (5000 × g, 15 min). The supernatant was extracted three times with diethyl ether/ethyl acetate (1:1, v/v). The combined extract was evaporated to dryness at 30°C. Dried conjugated and bound phenolics were dissolved separately in 1 mL of methanol and stored at −4°C until analysis.

#### HPLC analysis

Individual phenolic acids compounds in the wheat extracts were analyzed using a Waters 2695 high-performance liquid chromatograph (HPLC) equipped with a DIONEX AD25 Absorbance Detector and a Welch Ultimate AQ-C18 (250 × 4.60 mm, 5 μm) column (Waters, Milford, USA). The mobile phase, containing a gradient of solvent A (water containing 1% [v/v] HAc) and solvent B (100% methanol), was used at a flow rate of 0.6 mL/min. The total run time was 65 min and the gradient program was as follows: 20% B for 18 min, 27% B for 15 min, 20% B for 15 min, and 27% B for 17 min. The time of post-run (for reconditioning) was 5 min. The injection volume was 20 μL. Detection was performed at 280 nm using the absorbance detector. Phenolic acids in samples were identified and quantified based on comparisons with chromatographic retention times and areas of external standards. The following phenolic acids were quantified: ferulic acid, *p*-coumaric acid, vanillic acid, syringic acid, and caffeic acid.

### Determination of antioxidant activity using the frap assay

The FRAP (Ferric Reducing Ability of Plasma) assay was performed as reported by Benzie and Strain (1990), with slight modifications. Briefly, 0.5 mL of diluted extracts or standard solutions was mixed with 1.8 mL TPTZ solution (25 mL of 0.3 M acetate buffer, 2.5 mL of 10 mM TPTZ, and 2.5 mL of 20 mM FeCl_3_). The mixture was conditioned at 37°C for 10 min. Absorbance was measured at 593 nm. FRAP values are expressed in terms of FeSO_4_ equivalents (mM FeSO_4_/g dry weight).

### Determination of antioxidant activity using the ABTS^+^ assay

The ABTS^+^ (2, 2-azino-bis-(3-ehylbenzothiazoline-6-sulfonic acid) assay was performed according to the method of Shen et al. (2009). ABTS^+^ (3.9 mL) was added to the extract (0.1 mL) and mixed thoroughly. The mixture was incubated at room temperature for 6 min, after which the absorbance was immediately measured at 734 nm. Trolox standard solution in 80% ethanol was prepared and assayed under the same conditions. The results are expressed in terms of Trolox equivalents (μmol TE/g dry weight).

### Primers design, RNA extraction, and real-time PCR

Phenylalanine ammonia lyase (PAL), coumaric acid 3-hdroxylase (C3H), cinnamic acid 4-hydroxylase (C4H), 4-coumarate CoA ligase (4CL), and caffeic acid/5-hydroxyferulic acid O-methyltransferase (COMT) are the key enzymes involved in phenolic acid biosynthesis. Primers for phenolic acid biosynthesis gene, *TaPAL1, TaPAL2, TaC3H1, TaC3H2, TaC4H, Ta4CL1, Ta4CL2, TaCOMT1*, and *TaCOMT2*, were designed based on the corresponding genomic sequences of wheat listed in NCBI (http://www.ncbi.nlm.nih.gov). The primer sets used to amplify the phenolic acid biosynthesis genes and the actin gene (housekeeping gene) are listed in Table 1. Twenty immature grains from different plants at 7 DAA and 35 DAA of grain development were used for RNA extraction at each sampling time point, whereas 10 grains were used at 14, 21, and 28 DAA. Three replicates of each grain sample were used as biological replicates for each stage of development for the three wheat varieties. Total RNA was isolated from wheat samples using TRIzol Reagent (Thermo Fisher Scientific) according to the manufacture's instructions. Three PCRs per sample were performed to obtain the average expression levels of technical replicates. Expression analysis of genes was performed using SYBR Premix ExTaq (TaKaRa Biotechnology [Dalian] Co. Ltd.) according to the manufacturer's instructions. Relative quantity was calculated using the 2^−ΔΔCT^ method (Livak and Schmitthen, 2001). Gene expression values were normalized to the expression of Yumai49-198 on day 7 after anthesis (**Figure 2**).

**Table 1:** **Names and sequences of oligonucleotide primers used for gene amplification in this study**.

**Gene**	**Primer**	**Sequence (5′−3′)**	**Reference sequence**
*Ta4CL1*	4CL1-F	TACAACAACGGGCTGACCT	CJ962785
	4CL1-R	CCTTGAAGCCGTAGTCCAG	
*Ta4CL2*	4CL2-F	GAGTCCACAAAGAACACCA	BE403254
	4CL2-R	TGATTATCTCCTTGAGCCTG	
*TaC4H1*	C4H1-F	CAGCCTCCACATCCTCAAG	CK157495
	C4H1-R	CTTAGGACGAGCGAACAATC	
*TaC3H1*	C3H1-F	ATTGACGAAGAAGGGCAG	CD895891
	C3H1-R	GGACACAGCCATCTCAAGT	
*TaC3H2*	C3H2-F	TAGCAAGCATCATTTCGTG	AJ585990
	C3H2-R	TGTCCAACTCCTCTTGTAGC	
*TaCOMT1*	COMT1-F	ACGCTGCTCAAGAACTGCT	DQ223971
	COMT1-R	CGGGTTCACAGGCAGGAT	
*TaCOMT2*	COMT2-F	CAGGGAGAGGTACGAGAG	AY226581
	COMT2-R	GTAGATGTAAGTGGTCTTGATG	
*TaPAL1*	PAL1-F	CACCACCCTGGACAGATTG	AY005474
	PAL1-R	TGAGGCGAAGTGCGGAG	
*TaPAL2*	PAL2-F	GAGCGTGAGATCAATTCCG	X99705.1
	PAL2-R	GCAGACCGTTGTTGTAGAAGT	

### Statistical analysis

Data were analyzed and evaluated using SPSS (Statistical Program for Social Science) software using one-way analysis of variance (ANOVA). Differences between wheat varieties were evaluated using Fisher's least significant difference (LSD) test; *p* < 0.05 was considered to be statistically significant. Fisher's LSD test was also used to distinguish differences in gene expression levels between grains at different times during development. The genes were categorized into three groups according to their relative expression levels, as follows: High levels at the early stage: the expression levels were significantly higher at 7 or 14 DAA than at the other stages; High levels at the later stage: the expression levels were significantly higher at 35 DAA than that at the other stages; High levels at both the early and later stage: the expression levels were significantly higher at the early and later stage than at the other stages.

## Results

### Total phenolic content and antioxidant activity

As shown in Table 2, there were significant differences in TPC and antioxidant activity (ABTS^+^ and FRAP) among the six genotypes examined. Purple wheat had the highest TPC (1489.8 and 1525.1 μg/g, respectively), while white wheat had the lowest TPC. However, no significant difference in TPC was observed between white and red wheat. Similar results were also found for ABTS^+^ activity. The two purple wheat varieties had the highest ABTS^+^ activity (14.35 and 14.08 μmol TE/g), while white wheat cv. Zhengmai366 had the lowest ABTS^+^ activity (12.67 μmol TE/g). Purple wheat cv. Zhouheimai1 had the highest FRAP activity, whereas white wheat had the lowest values (10.55 and 10.79 μmol FeSO_4_/g, respectively). There were significant differences in FRAP activity between Zhouheimai1 and other varieties. However, no significant difference in FRAP activity was observed between the two white wheat varieties and the red wheat variety Yangmai15.

**Table 2:** **Variances of total phenolic content and antioxidant activity among wheat varieties with different grain colors^a^^,^^b^^,^^c^**.

**Grain color**	**Variety**	**TPC (FAE μg/g)**	**ABTS^+^(μmol TE/g)**	**FRAP (μmol FeSO_4_/g)**
White	Yumai49-198	1147.0c ± 72.5	12.80c ± 0.27	10.55d ± 0.84
	Zhengmai366	1208.7c ± 33.0	12.67c ± 0.25	10.79d ± 0.32
Red	Yangmai15	1256.9bc ± 95.1	12.72c ± 0.04	11.39cd ± 0.53
	Yangmai22	1347.6abc ± 65.5	13.00bc ± 0.18	12.29bc ± 0.62
Purple	Zhouheimai1	1489.8ab ± 22.9	14.35a ± 0.55	15.73a ± 1.53
	Shandongzimai1	1525.1a ± 110.8	14.08a ± 1.50	13.56b ± 0.48

a Values are expressed as mean ± standard deviation.

b Means in the same column followed by different letters are significantly different (p < 0.05).

c FAE stand for ferulic acid equivalents.

### Phenolic acid compounds

As shown in Tables 3, 4, bound phenolic acids comprised 87.1–90.6% of total phenolic acids. Ferulic acid was the predominant bound phenolic form, accounting for 91.1–93.4% of total bound phenolics. Contents of soluble ferulic acid and syringic acid were comparable (from 20.94 to 37.31 μg/g). Caffeic acid (in both bound phenolic and soluble form) had the lowest value (0.10–1.78 μg/g). Additionally, the patterns of variation in phenolic acid contents differed among genotypes. The bound ferulic acid and vanillic acid contents in purple wheat varieties were significantly higher than those of white and red wheat. A significant difference in caffeic acid levels was also observed between the two purple wheat varieties. Significantly lower levels of bound *p*-coumaric acid were detected in the white-grained genotype, whereas unlike the other phenolic acids, no significant difference was observed between red and purple-grained types. A similar pattern was observed for soluble ferulic acid and vanillic acid, with purple and red wheat containing higher levels of these compounds than white wheat. A significant difference in soluble caffeic acid levels was detected between the purple wheat varieties and the other wheat varieties. The highest level of soluble syringic acid was detected in Yangmai22 (34.44 μg/g,) whereas the lowest level was detected in white wheat variety Yumai49 (20.94 μg/g). No significant difference in *p*-coumaric acid levels was observed among purple, red, and white wheat.

**Table 3:** **Variances of bound phenolic compound levels among wheat varieties with different grain colors^a^^,^^b^**.

**Grain color**	**Variety**	**Ferulic acid (μg/g)**	***p*-coumaric acid (μg/g)**	**Syringic acid (μg/g)**	**Vanillic acid (μg/g)**	**Caffeic acid (μg/g)**	**Total phenolic acid (μg/g)**
White	Yumai49-198	573.3b ± 66.0	14.74b ± 1.81	9.63a ± 3.58	18.42b ± 0.74	0.11c ± 0.01	616.1c ± 145.7
	Zhengmai366	563.9b ± 54.3	13.02b ± 6.75	9.40a ± 3.45	16.74b ± 1.73	0.11c ± 0.02	603.1c ± 145.2
Red	Yangmai15	646.8b ± 89.6	24.14a ± 2.02	8.04a ± 1.46	23.44b ± 2.18	0.10c ± 0.02	702.5b ± 81.1
	Yangmai22	701.4b ± 42.2	27.63a ± 3.85	9.58a ± 1.42	28.41b ± 3.11	0.13c ± 0.04	767.1b ± 42.5
Purple	Zhouheimai1	823.8a ± 82.6	25.80a ± 7.29	9.17a ± 1.43	44.86a ± 10.95	0.34a ± 0.07	903.9a ± 65.6
	Shandongzimai	838.7a ± 83.0	28.46a ± 4.32	8.69a ± 0.53	41.14a ± 7.59	0.26b ± 0.03	917.2a ± 79.3

a Values are expressed as mean ± standard deviation.

b Means in the same column followed by different letters are significantly different (p < 0.05).

**Table 4:** **Variances of soluble phenolic compound levels among wheat varieties with different grain colors^a^^,^^b^**.

**Grain color**	**Variety**	**Ferulic acid (μg/g)**	***p*-coumaric acid (μg/g)**	**Syringic acid (μg/g)**	**Vanillic acid (μg/g)**	**Caffeic acid (μg/g)**	**Total phenolic acid (μg/g)**
White	Yumai49-198	21.88c ± 4.72	10.63a ± 3.91	20.94c ± 1.15	13.69c ± 3.62	0.80b ± 0.18	67.94b ± 4.22
	Zhengmai366	25.75c ± 3.87	11.24a ± 3.58	24.75bc ± 4.53	12.65c ± 0.95	0.97b ± 0.32	75.36b ± 10.43
Red	Yangmai15	35.85a ± 5.24	10.99a ± 3.82	30.21ab ± 4.37	23.77ab ± 3.80	0.85b ± 0.25	101.67a ± 14.29
	Yangmai22	32.39ab ± 5.23	22.07a ± 8.86	34.44a ± 4.55	24.14ab ± 7.40	0.61b ± 0.19	113.66a ± 22.91
Purple	Zhouheimai1	33.73ab ± 3.76	8.20a ± 1.06	25.61bc ± 2.01	29.21a ± 1.96	1.43a ± 0.37	98.19a ± 2.99
	Shandongzimai	37.31a ± 2.11	15.61a ± 5.39	23.16bc ± 3.86	17.85b ± 4.39	1.78a ± 0.63	95.71a ± 11.12

a Values are expressed as mean ± standard deviation.

b Means in the same column followed by different letters are significantly different (p < 0.05).

### Accumulation of phenolic acids in developing seeds

**Figure 1:**
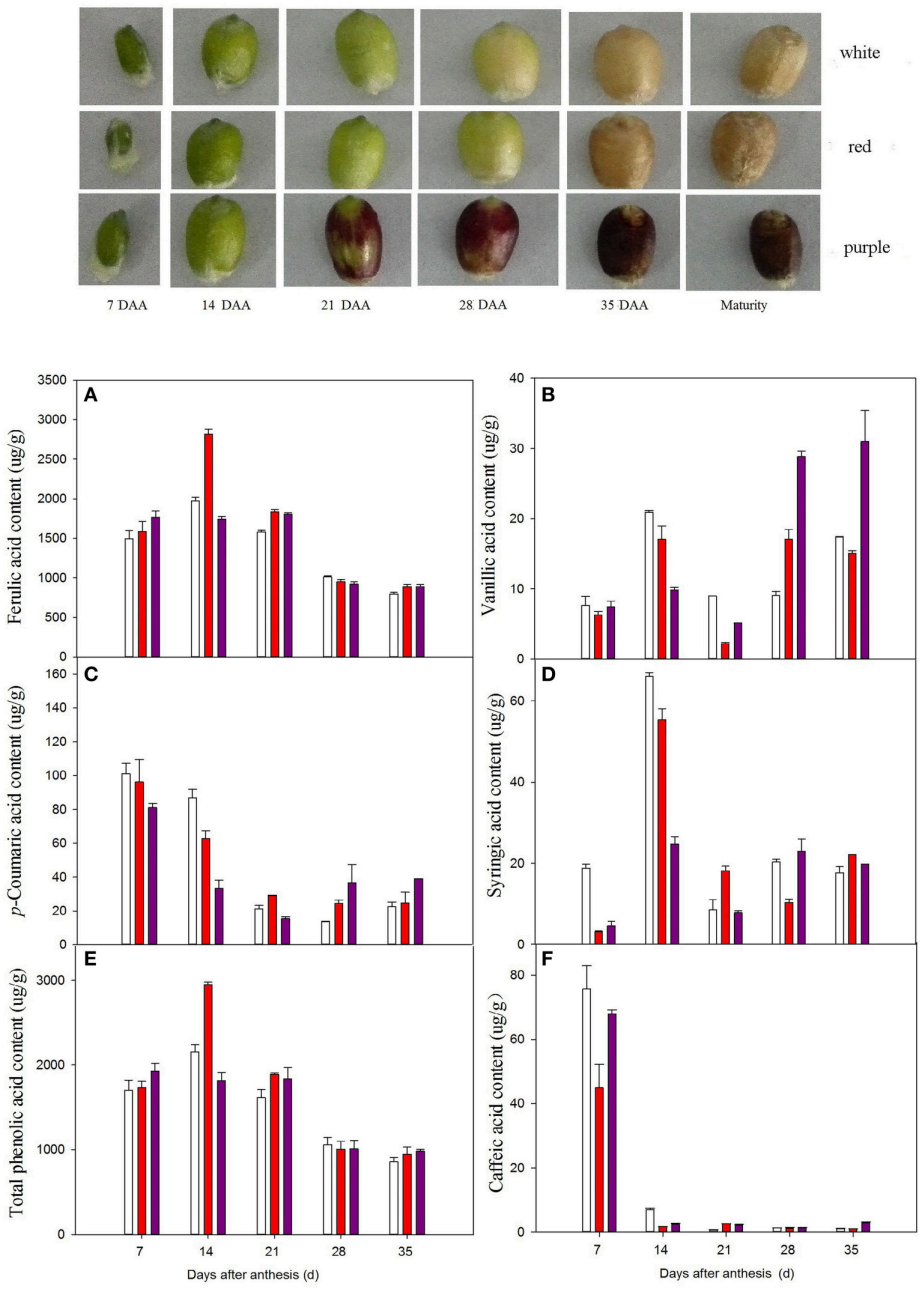
**Variance of bound phenolic compound of different wheat varieties during grain filling stage**. 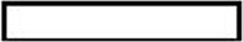, 
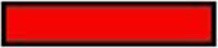
, and 
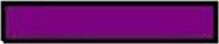
 stand for white wheat Yumai49-198, red wheatYangmai22 and purple wheat Zhouheimail, respectively. Panels (**A–F**) stand for ferulic acid, vanillic acid, *p*-coumaric acid, syringic acid, total phenolic acid, and caffeic acid, respectively.

Bound phenolics are the major phenolics in wheat. We therefore measured the accumulation of bound phenolics in wheat grains during development. Three different patterns of variation in phenolic acid contents were observed during grain filling (Figure 1). The levels of total bound phenolics (Figure 1E), ferulic acid (Figure 1A), and syringic acid (Figure 1D) peaked at 14 DAA. The levels of *p*-coumaric acid (Figure 1C) and caffeic acid (Figure 1F) reached a maximum at 7 DAA, followed by a decline. Specifically, caffeic acid levels ranged from 45.07 to 77.98 μg/g in various wheat varieties at 7 DAA and from 0.59 to 3.50 μg/g at 35 DAA. Vanillic acid levels gradually increased during grain filling and peaked near ripeness (35 DAA). Red wheat had the highest ferulic acid contents at 14 DAA. By contrast, white wheat had the highest levels of vanillic acid, *p*-coumaric acid, and syringic acid before 21 DAA, followed by red wheat and purple wheat. At 7 DAA, the levels of caffeic acid were highest in white wheat, followed by purple wheat and red wheat. However, the levels of phenolic acids at the middle and later stages of development (28 DAA and 35 DAA) were highest in purple wheat.

### Expression of phenolic acid biosynthesis genes in developing seeds

**Figure 2:**
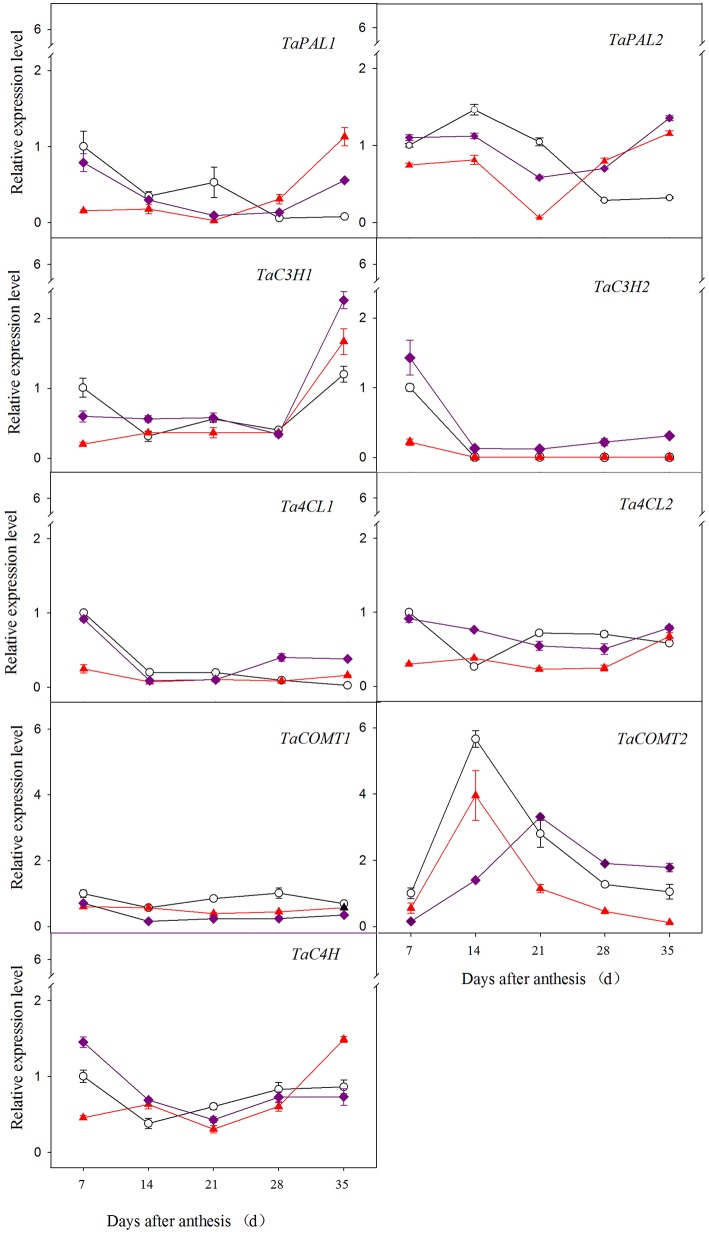
**Relative expression level of phenolic acid biosynthesis genes in different wheat grain during grain development**. 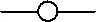, 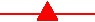, and 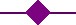 stand for white wheat Yumai49-198, red wheat Yangmai22, and purple wheat Zhouheimail, respectively.

As shown in Figure 2, the genes exhibited different expression patterns among wheat varieties across developmental time. In red and purple wheat, *TaPAL1* and *TaPAL2* were relatively highly expressed both at the early development stage (7 DAA or 14 DAA) and at the later stage (35 DAA) compared to the middle stage, while in white wheat, these genes were only highly expressed at the early stage. For example, the relative expression level of *TaPAL1* of purple wheat at 7 DAA and 35 DAA was 8.10- and 6.11-fold greater than that at 21 DAA, respectively. Two genes (*TaC3H1* and *TaC3H2*) exhibited opposite expression patterns during grain development; *TaC3H1* exhibited the highest expression at 35 DAA, whereas *TaC3H2* exhibited the highest expression at 7 DAA. In all three types of wheat, the expression of *Ta4CL1* decreased with increasing grain development, with the highest value observed at 7 DAA. In red and purple wheat, the expression of *Ta4CL2* was relatively high at both the early and later stage of grain development, whereas its expression peaked at 7 DAA in white wheat. The expression level of *TaCOMT1* initially declined, followed by an increase over the course of grain development, except for white wheat exhibiting decline tendency from 28 DAA to 35 DAA. The relative expression level of *TaCOMT1* of red wheat and purple wheat at 35 DAA was 1.46- and 1.47-fold greater than that at 21 DAA, respectively. *TaCOMT2* expression peaked at 14 DAA or 21 DAA for all three wheat varieties. Similar expression patterns were observed for *TaC4H* in all wheat varieties examined, with higher expression level observed at both 7 DAA (or 14 DAA) and 35 DAA than at the other stage.

**Figure 3:**
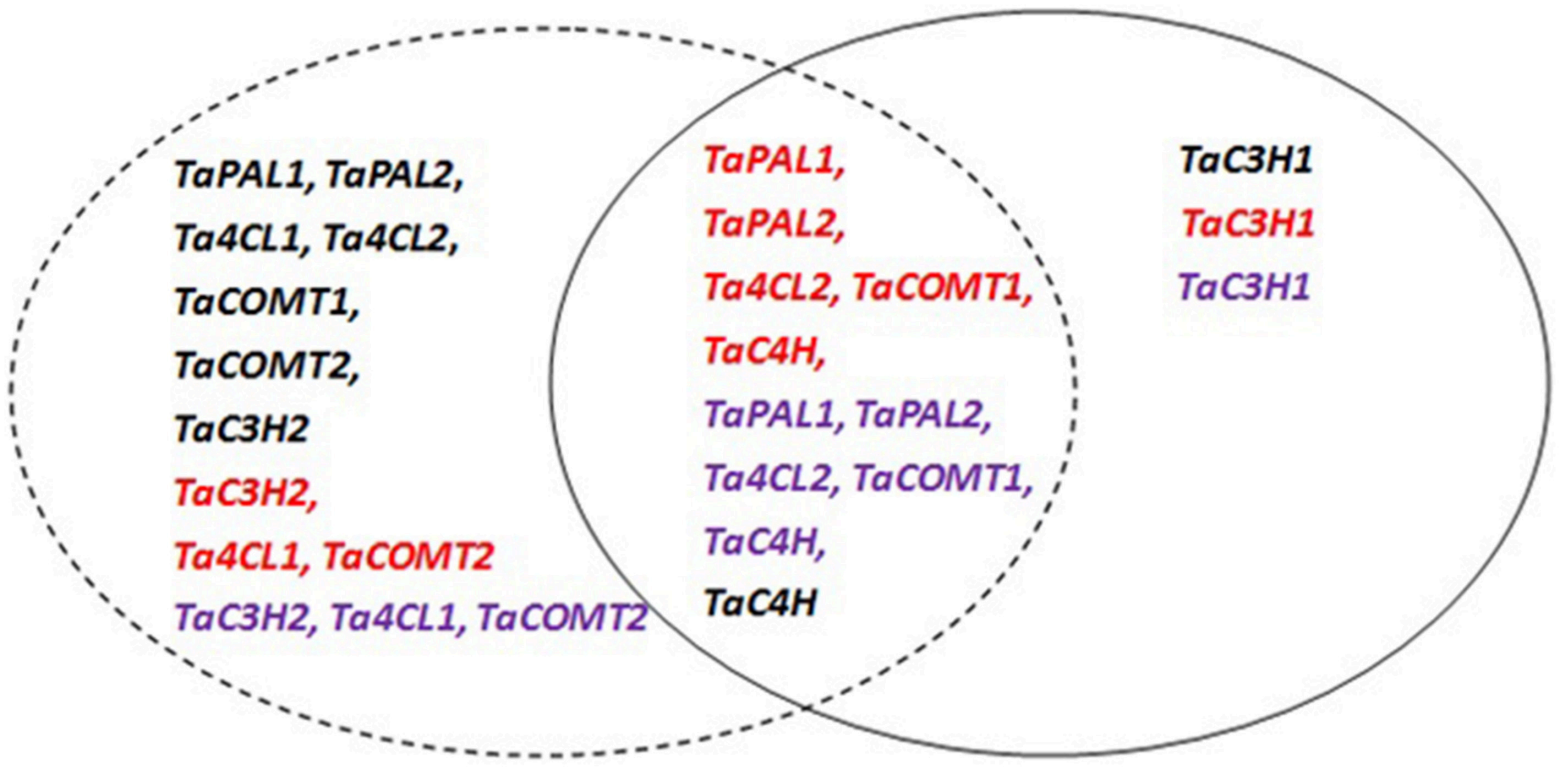
**Groups of wheat genes involved in phenolic acids synthesis in the grain according to their individual expression patterns during the grain development period**. Black, red, and purple colors stand for white, red and purple wheat, respectively. The dotted circle stand for higher gene expression level at early development stage, and solid circle stand for higher expression level at later stage.

According to the above results and the significant differences in gene expression levels among sampling times (data not shown), the expression profiles of wheat phenolic acid biosynthesis genes during grain development were divided into three groups (Figure 3). The first group is characterized by high expression levels during early grain development, as exhibited by *TaPAL1, TaPAL2, Ta4CL1, Ta4CL2, TaCOMT1, TaCOMT2*, and *TaC3H2* in white wheat and *TaC3H2, Ta4CL1*, and *TaCOMT2* in red and purple wheat. The second group, which includes *TaC3H1*, was more highly expressed at the later stage of grain development in all three types of wheat. The third group had higher expression levels during both early and later grain development, including *TaC4H* in white wheat and *TaPAL1, TaPAL2, Ta4CL2, TaCOMT1*, and *TaC4H* in red and purple wheat. Most white wheat genes belong to the first group, while most red and purple wheat genes belong to the third group.

Among gene family members, *TaPAL2* was more highly expressed than *TaPAL1*, and *TaCOMT2* was more highly expressed than *TaCOMT1*. *TaC3H1* was more highly expressed than *TaC3H2* at 35 DAA, which indicates that *TaC3H1* may play a more important role than *TaC3H2* at later grain development stages. Among wheat varieties with different grain colors, phenolic acid biosynthesis genes exhibited different expression patterns during grain development. During early development, five genes, including *TaPAL1, TaPAL2, Ta4CL1, Ta4CL2*, and *TaC3H1*, had the highest expression levels in white wheat, followed by purple wheat or red wheat. *TaC3H2* and *TaC4H* had the highest expression levels in purple wheat during early development (before 14 DAA). However, most of the genes had similar expression patterns at later stages; the expression of these genes increased in purple wheat beginning at 28 DAA and reached higher levels than those observed in white wheat at 35 DAA. For example, there was a 3.7-fold difference in *TaPAL1* expression between purple and white wheat at 35 DAA. Similarly, at 35 DAA, the expression level of *TaCOMT2* in purple wheat was 1.78-fold greater than that of white wheat. Six genes, including *TaPAL2, TaC3H1, TaC3H2, Ta4CL1, Ta4CL2*, and *TaCOMT2*, had the highest expression levels in purple wheat at 35 DAA. As mentioned above, purple wheat had significant higher phenolic acids content than white wheat in maturity grain, including bound phenolic acids (ferulic acid, *p*-coumaric acid, vanillic acid, caffeic acid, total phenolic acid), and soluble phenolic acids (ferulic acid, vanillic acid, caffeic acid, total phenolic acid). These results suggested that gene expression in purple wheat at later developmental stages may play a more important role in determining purple grain phenolic acids contents.

## Discussion

### Phenolic contents among different wheat cultivars

Phenolics are biologically active substances in wheat grains. Regular intake of these antioxidants can reduce the risk of cardiovascular disease and certain cancers (Lutsey et al., 2007). In recent years, pigmented wheat has become popular due to its high free radical scavenging ability and phenolic contents. In this study, we found that two purple wheat varieties had significantly higher TPC than white wheat, which is in agreement with the findings of Li et al. (2005). No significant difference in TPC was observed between purple and red wheat, whereas the total phenolic acid content (bound) in purple wheat was significant higher than that in red wheat. The main reason for this increase is that there were other phenols included in the TPC value (tested by Folin Ciocalteu reagent) in addition to the phenolic acid compound measured in this study. Another possible reason involves the interaction between genotype and growing area. Indeed, the growing area, crop year, genotype, and their interaction have considerable effects on grain TPC (Martini et al., 2015). Insoluble-bound phenolic acids fractions are of particular importance due to their ability to prevent the oxidation of biologically important molecules in the colon (Andreasen et al., 2001). We found that purple wheat had significantly higher total bound phenolic acids contents than the other types, followed by red and white wheat. These results suggest that purple wheat, which is rich in antioxidants, especially total bound phenolic acids, should be used in health food production.

### Phenolic accumulation during seed development

Wheat grains change as they develop, including changes in the surface area-to-volume ratio, dry matter content, and antioxidant content. McCallum and Walker (1990) reported that the levels of soluble phenolics per seed peak just prior to the appearance of mature grain color, but the concentration of phenolics (expressed on a dry weight basis) declines during maturation. In the current study, the levels of most phenolic acids in wheat grains (expressed on a dry weight basis) declined during grain development, and the highest values were detected at the early stage (7 DAA or 14 DAA). Similar results were also obtained in a study of developing rice grains, in which the TPC (mg/100 g) values for white rice and red rice were significantly higher at 1 week after flowering than at 3 weeks after flowering (Shao et al., 2014). The reduced levels of these substances may result from the dry matter dilution effect (Knievel et al., 2009). This observation may also be attributed to changes in assimilate availability (Bustos et al., 2012). Starch synthesis primarily occurs in the middle and later grain filling stages. Thus, the reduced substrate supply during wheat grain development may affect the biosynthesis of phenolic acids. Lewis et al. (1999) speculated that the presence of sucrose is necessary for polyphenol biosynthesis during potato tuber development. In the current study, the fluctuation in vanillic acid levels differed from that of other phenolic acids, with the highest levels detected during later development (35 DAA). A peak in vanillic acid levels at maturity was also detected in black rice (Shao et al., 2014). White wheat had relatively high phenolic acids levels compared to purple wheat at the early stage of grain development (before 14 DAA), whereas purple wheat had relatively high phenolic acids levels from the middle stage of grain development (after 28 DAA) to full grain maturity. These results suggest that agronomic treatments to increase phenolic acid levels may be more effective at the middle and later grain filling stages than at the early stage. Indeed, application of Mg fertilizer to purple wheat close to physiological maturity increased the anthocyanin content by 65% (Bustos et al., 2012). In addition, André et al. (2009) suggested that the level of a polyphenol compound is regulated by the rates of its biosynthesis and degradation. Bustos et al. (2012) reported that high levels of polyphenols (including anthocyanins) in wheat grains could be ascribed to the reduced degradation of anthocyanins. The degradation of phenolic acids in wheat grains and the conversion between phenolic acid fractions require further study.

### Gene expression and phenolic accumulation

Phenolic acids in plants are primarily derived from the phenylpropanoid biosynthetic pathway. PAL functions in the entry point of the phenolic acid pathway by catalyzing phenylalanine to cinnamic acid (Nair et al., 2004). PAL, C3H, C4H, 4CL, and COMT are the major enzymes in the phenolic acids biosynthesis pathway (Supplementary Figure S1); their action leads to the production of different phenolic acid compounds. In the current study, we found that the expression of genes involved in phenolic acids biosynthesis varied throughout wheat grain development. Similar results were previously obtained for other plants. For example, Fock-Bastide et al. (2014) found that *VpPAL1* was gradually upregulated during *Vanilla planifolia* pod development, reaching maximum expression levels at maturity, while genes encoding the enzymes 4HBS, C4H, OMT2, and OMT3 were not significantly upregulated after the fourth month post-pollination. In the present study, we found that the genes examined could be classified into three groups according to the time at which their expression peaked. The expression patterns of the genes corresponded to the accumulation patterns of phenolic acid compounds. The first group of genes, which were highly expressed at the early stage of grain development, may be related to the accumulation of ferulic acid, *p*-coumaric, syringic, and caffeic acid. *TaC3H1* (the second group) was more highly expressed at later grain filling stages, which may be related to the accumulation of vanillic acid. Genes in the third group were highly expressed at both early and later stages of grain development; these genes may play important roles in the accumulation of all phenolic acids examined. A relationship between the expression levels of phenolic acids biosynthesis genes and phenolic acid levels was also reported by Jiang et al. (2013).

The expression patterns of two *TaPAL* genes in red and purple wheat revealed that these genes are related to the accumulation of all phenolic acid fractions examined. This finding helps confirm that the enzyme PAL is involved in the first committed step in the phenyl propanoid pathway and is therefore involved in the biosynthesis of polyphenol compounds. McCallum and Walker (1990) and André et al. (2009) also reported that PAL activity and *PAL* expression are closely related to the accumulation of phenolic acid. Indeed, we noticed that in white wheat, *TaPAL* was relatively highly expressed only at the early stage of grain development, and the vanillic acid content in white wheat was also relatively high at 7 DAA; these results help to confirm that PAL is related to the accumulation of vanillic acid. Phenylalanine is processed into *p*-coumaric acid by C4H during the early steps of the phenylpropanoid pathway. Then, *p*-coumaric acid is metabolized to ferulic acid by C3H and COMT (Boerjan et al., 2003). In this study, we found that the expression of *TaC4H* was related to *p*-coumaric acid accumulation, and *Ta4CL, TaCOMT*, and *TaC3H2* expression was related to the accumulation of ferulic acid. Additionally, white wheat had higher phenolic acid contents and relatively high gene expression at the early stage, while purple wheat had the highest phenolic acid contents and gene expression levels at later stages. These results confirm that the expression of phenolic acid biosynthesis genes is positively correlated with phenolic acid accumulation.

Many genes are present in multigene families, with several copies present in the genome. In the current study, *TaPAL2* was expressed at higher levels than *TaPAL1* during grain development. Gayoso et al. (2010) reported that of the six *PAL* genes examined in tomato roots, *PAL2* was the most highly expressed, followed by *PAL3, PAL4*, and *PAL6*. Similar gene expression profiles were observed for *TaCOMT, Ta4CL*, and *TaC3H* in the present study. Two *BnC4H* and two *Cs4CL* genes are present in rapeseed and tea, respectively (Jiang et al., 2013; Qu et al., 2013). These findings indicate that different genes in multigene families play different roles in phenolic acids biosynthesis. Further studies are needed to identify the major genes involved in the biosynthesis of specific phenolic acids and to explore the potential of manipulating the expression of these genes to improve grain antioxidant contents.

In conclusion, in mature seeds, purple wheat had the highest total phenolic contents and antioxidant activity, while white wheat had the lowest values. White wheat and red wheat had the highest phenolic acid contents during early seed development, while the levels of phenolic acids during later development were highest in purple wheat. Nine phenolic acid biosynthesis genes exhibited three distinct expression patterns during grain filling, which were in accordance with the accumulation patterns of different phenolic acid compounds. The expression patterns of genes in the developing grain are likely responsible for the accumulation patterns of phenolic compounds in the grain. Further studies are needed to uncover the functions of various genes involved in the biosynthesis of different phenolic acid compounds in wheat grains.

## Author contributions

The work presented here was carried out in collaboration between all authors. CW and TG defined the research theme and co-designed experiments, and discussed analyses. DM designed methods, experiments, and wrote this paper. YL carried out the laboratory experiments and analyzed the data. HD and HQ co-worked on associated data collection and their interpretation. YX and JZ co-worked on experimetns design and obtaining test data. All authors have contributed to, seen and approved the manuscript.

## Funding

This project was funded by the Special Funds for Agro-scientific Research in the Public Interest (201203031), the Science and Technology Support Program (2015BAD26B00), and the Scientific and Technological Project of Henan Province (152102110067).

### Conflict of interest statement

The authors declare that the research was conducted in the absence of any commercial or financial relationships that could be construed as a potential conflict of interest.
