# Supplementary material for: Accumulation of Phenolic Compounds and Expression Profiles of Phenolic Acid Biosynthesis-Related Genes in Developing Grains of White, Purple, and Red Wheat
Source: Front Plant Sci. 2016 Apr 22;7:528. doi: 10.3389/fpls.2016.00528 (PMC4840273; doi:10.3389/fpls.2016.00528)
Supplement: Supplementary file 1 [file Table1.DOCX]

**Supplementary**

Table S1 Environmental conditions at the growing location during wheat growth period

| Date(Month) | Temperature (^0^C) | | | Rainfall(mm) | Accumulation  irradiation time (h) |
| --- | --- | --- | --- | --- | --- |
|  | Max | Min | Mean |  |  |
| October | 32.9 | 4.4 | 17.2 | 27.2 | 166.8 |
| November | 23.4 | -0.1 | 9.7 | 32.3 | 172.9 |
| December | 17.4 | -6.1 | 3.6 | 0.0 | 136.9 |
| January | 16.3 | -6.9 | 4.0 | 0.1 | 126.6 |
| February | 14.6 | -8.8 | 2.7 | 24.3 | 70.2 |
| March | 29.2 | 0.8 | 13.0 | 6.8 | 186.0 |
| April | 29.4 | 6.5 | 17.1 | 56.4 | 154.5 |
| May | 39.4 | 8.1 | 24.1 | 57.6 | 242.3 |
