# Supplementary figures and images for: Accumulation of Phenolic Compounds and Expression Profiles of Phenolic Acid Biosynthesis-Related Genes in Developing Grains of White, Purple, and Red Wheat
Source: Front Plant Sci. 2016 Apr 22;7:528. doi: 10.3389/fpls.2016.00528 (PMC4840273; doi:10.3389/fpls.2016.00528)

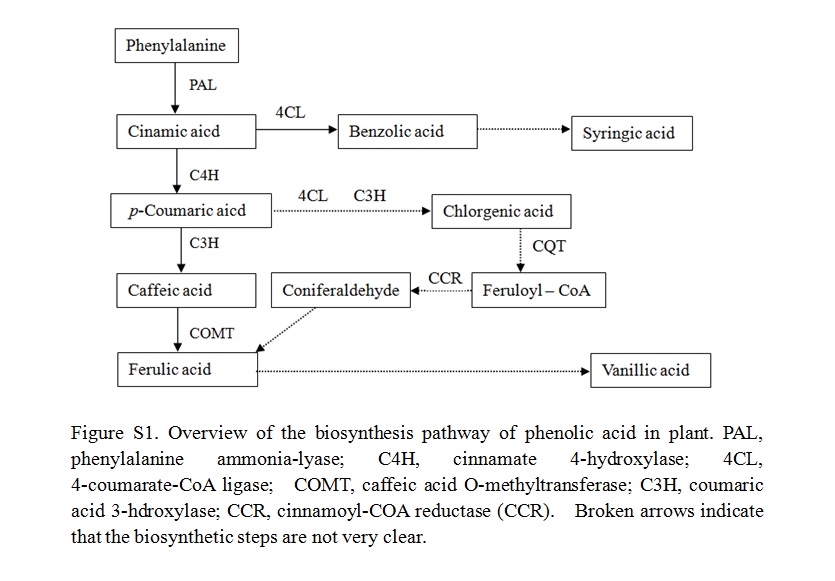

Supplement: Supplementary file 2 [file Image1.JPEG]
